# A Novel Fatty Acid-Binding Protein-Like Carotenoid-Binding Protein from the Gonad of the New Zealand Sea Urchin *Evechinus chloroticus*

**DOI:** 10.1371/journal.pone.0106465

**Published:** 2014-09-05

**Authors:** Jodi Pilbrow, Manya Sabherwal, Daniel Garama, Alan Carne

**Affiliations:** 1 Department of Biochemistry, University of Otago, Dunedin, New Zealand; 2 Centre for Protein Research, Department of Biochemistry, University of Otago, Dunedin, New Zealand; 3 Monash Institute of Medical Research-Prince Henry's Institute, Monash University, Melbourne, Victoria, Australia; Russian Academy of Sciences, Institute for Biological Instrumentation, Russian Federation

## Abstract

A previously uncharacterized protein with a carotenoid-binding function has been isolated and characterized from the gonad of the New Zealand sea urchin *Evechinus chloroticus*. The main carotenoid bound to the protein was determined by reversed phase-high performance liquid chromatography to be 9′-*cis*-echinenone and hence this 15 kDa protein has been called an echinenone-binding protein (EBP). Purification of the EBP in quantity from the natural source proved to be challenging. However, analysis of EBP by mass spectrometry combined with information from the *Strongylocentrotus purpuratus* genome sequence and the recently published *E. chloroticus* transcriptome database, enabled recombinant expression of wild type EBP and also of a cysteine61 to serine mutant that had improved solubility characteristics. Circular dichroism data and *ab initio* structure prediction suggests that the EBP adopts a 10-stranded β-barrel fold consistent with that of fatty acid-binding proteins. Therefore, EBP may represent the first report of a fatty acid-binding protein in complex with a carotenoid.

## Introduction

For over 100 years the sea urchin has been established as an important biological model organism. A simple body plan and the relative ease of reproductive manipulation have made the sea urchin a popular animal for the study of reproduction and development [1], [2], [3]. In addition to this service to biology, sea urchins have also provided a valuable food resource to coastal populations around the world since prehistoric times [4]. Sea urchins continue to be fished for their edible gonads (known as roe or uni), which are considered a culinary delicacy that is popular in parts of Asia, Europe and South America [4], [5].

The sea urchin *Evechinus chloroticus* (Kina) is endemic to coastal New Zealand waters, from which it is harvested on a small scale to supply gonads to domestic and potentially, international markets. Although the relatively large size of *E. chloroticus* gonads is a desirable market attribute, current export success is limited by variability in gonad color between animals [5], [6]. Export market values of the product are dependent upon taste and texture but with particular importance placed on color for attractive culinary presentation [7]. Yellow/orange colored gonads are the most desirable [7] and can attain up to US$400/Kg [8], however undesirable shades such as brown and black are also common amongst *E. chloroticus* [5], [6], which adversely affects the market value.

Sea urchin gonad color appears primarily to be due to carotenoid pigment molecules deposited within the tissue [9], [10], [11], [12], [13]. Other than two notable exceptions in the American cockroach [14] and pea aphid [15], animals are unable to synthesize carotenoids *de novo* [16]. However, sea urchins are able to obtain precursor carotenoids form the diet that may be modified in the viscera and then transported to the gonads [12], [17], [18]. The assimilation of carotenoids in the gonads suggests that they play an important role in sea urchin reproduction. In support of this hypothesis, it has been observed that gonad carotenoid concentrations fluctuate in accordance with the annual reproductive cycle [9], [19]. Furthermore, it has been demonstrated that carotenoids are incorporated into oocytes and may provide anti-oxidant protection to spawned eggs and juveniles during early development [20], [21], [22]. The interconnected role of carotenoids in sea urchin reproduction and gonad coloration provides both a biological and economical incentives to gain a better understanding of carotenoid biochemistry in the sea urchin gonad.

Echinenone has been found to be the major carotenoid present in the gonads of many species of sea urchin, accounting for up to 85% of total carotenoid [9], [10], [11], [12], [13], [23]. Detailed analyses resolving geometric isomers have indicated that 9′-*cis*-echinenone (9′*Z*-β-echinenone) predominates over the all-*trans*-isomer (all-*E*-β-echinenone) [12], [24]. However, echinenone isomers represent only a small proportion of total gut carotenoids [12], [24], suggesting that they are selectively taken up and assimilated by the gonad. Such selectivity is likely to be mediated through interactions with specific carotenoid-binding proteins (CBP).

Carotenoids in complex with proteins (carotenoproteins) have been isolated from both prokaryotic and eukaryotic organisms. The well-researched involvement of carotenoids in light-harvesting processes has led to the identification of many carotenoprotein complexes from plants and photosynthetic microorganisms. Conversely the number of complexes identified from the animal kingdom is disproportionately low. A β-carotene-binding protein isolated from rat [25] and ferret liver [26] and a zeaxanthin-binding protein from human macula and retinal tissue [27], [28], [29], represents the exiguous information from vertebrates. Although a greater number of complexes have been reported from invertebrates, including several from marine animals, many have received very little additional characterization. Crustacyanin, the lobster carapace protein that forms a complex with astaxanthin, is one of few well characterized CBPs. Furthermore, crustacyanin remains the only CBP of animal origin for which a structure has been experimentally determined [30], [31]. The X-ray crystal structure of the holo-β-crustacyanin complex revealed a heterodimer of A_1_ and A_3_ crustacyanin subunits with two astaxanthin molecules interpenetrating the dimer interface [31].

The X-ray crystal structure of crustacyanin indicated that the complex belonged to the lipocalin family [30], [31], which also includes other CBPs from photosynthetic organisms [32], [33]. Lipocalin proteins form a sub-group of calycin protein family, which is characterized by a common tertiary structural fold; an 8 or 10 stranded β-barrel. The β-barrel fold forms a cup-shaped or “calyx” binding-pocket, which is able to accommodate a wide-variety of small hydrophobic ligands [34], [35]. In addition to lipocalins the calycin superfamily also encompasses avidins, triabins, metalloproteinase inhibitors and fatty acid-binding proteins (FABPs) [34], [36]. However, despite commonality of tertiary structural fold, members of the lipocalin sister families are not known to interact with carotenoids. FABPs are likely candidates for CBPs, as although they are named for their role as intracellular lipid carriers, FABPs are known to bind a diverse range of small hydrophobic ligands [37]. Furthermore, cellular retinol, retinal and retinoic acid binding protein classes of FABPs are specialist carriers of their respective carotenoid metabolites [38].

The accumulation of carotenoids in sea urchin gonads is likely to play an important role in reproduction and contributes to the coloration of the tissue. It appears that the assimilation of carotenoids is a selective process that is likely to be mediated through interactions with proteins. This research aimed to identify a carotenoid-protein complex from the gonad of the New Zealand sea urchin *Evechinus chloroticus*. A small protein, of approximately 15 kDa in size was identified and purified in complex with 9′-*cis*-echinenone from the gonad of *E. chloroticus*, and has therefore been called an echinenone-binding protein (EBP). Spectroscopic analysis of wild type recombinant EBP (apo-rEBP) and of a recombinant cysteine61 to serine mutant (apo-rEBP-C61S), in conjunction with *ab initio* secondary structure predictions and sequence analysis suggests that the protein belongs to the FABP sub-group of the calycin protein superfamily. Therefore the EBP may represent the first report of a FABP in complex with carotenoid.

## Materials and Methods

### Ethics statement

No ethical approval was required for the use of *E. chloroticus* for this research. However, sea urchin specimens were obtained and used for scientific research with permission from and with respect to the beliefs of the local Māori Iwi.

### Native purification

*E. chloroticus* specimens were provided by Campbell McManaway at Cando Fishing NZ, where the animals were dissected and tissues were stored at −20°C until required. The method for the purification of carotenoid-binding proteins (CBPs) from gonad tissue was adapted from Jouni and Wells [39]. Defrosted gonad tissue was weighed and blended with an equal w/v of homogenization buffer (0.05 M dibasic sodium phosphate, 50 mM sodium chloride pH 8.0, containing cOmplete-mini EDTA-Free protease inhibitor cocktail, Roche Applied Sciences, Penzberg, Germany) using a Sorvall Omni-Mixer (DuPont Instruments, CT, USA) at 2,500 rpm until homogeneous. The homogenate was centrifuged at 4°C and 8,000 *g* for 30 min and the supernatant fraction was removed and retained.

Delipidation of the sample was performed by density-adjusted ultra-centrifuging, based on protocols modified from Redgrave *et al*. [40] and Havel *et al*. [41] and Aviram [42]. The density of the supernatant was adjusted to 1.2 g.mL^−1^ by the addition of crystalline sucrose and then ultra-centrifuged at 4°C and 435,000 *g* for 1 h in a TL-100 ultra-centrifuge (Beckman, CA, USA). Following ultra-centrifuging the sample was extracted from beneath a lipid layer with a needle and syringe and then dialyzed at 4°C overnight against ion-exchange chromatography buffer A (0.05 M dibasic sodium phosphate, pH 8.0).

The dialyzed sample was passed through a bed (150 × 15 mm) of DEAE Sepharose resin (pre-equilibrated with anion-exchange buffer A), under gravity flow, to remove additional lipid from the sample. Bound proteins were eluted with 0.5 M NaCl and collected in 3 mL fractions. Yellow-colored fractions were buffer exchanged to ion-exchange chromatography buffer A and re-concentrated using a Vivaspin 10 kDa MWCO centrifugal-concentrator (GE Healthcare, Uppsala, Sweden). The sample was then extruded through a 0.45 µm PTFE filter (Sartorius, Goettingen, Germany) and 3 mL was injected onto a pre-equilibrated 5 mL HiTrap Q-Sepharose fast-flow column (GE Healthcare), connected to an ÅKTA-Explorer 900 FPLC (GE Healthcare). The column was washed with 25 mL of buffer A, at a flow-rate of 5 mL.min^−1^. Bound protein was eluted with a 0–100% gradient of buffer B (buffer A + 1 M NaCl), over 100 mL. Column effluent was monitored at 280 nm and 445 nm and 2 mL fractions were collected. Fractions with absorbance at both 280 nm and 445 nm were pooled from across multiple runs and re-concentrated.

Glycerol was added to the re-concentrated sample to achieve 20% v/v and then an aliquot was separated under non-denaturing and non-reducing conditions by 1D native-PAGE on a 7.5% v/v acrylamide, continuous Tris-glycine (pH 8.8) mini-gel, using a Mini-Protean 3 electrophoresis system (BioRad, CA, USA). Following electrophoresis, the yellow/orange band was excised, diced into 1 mm^3^ cubes and incubated on ice with 200 µL of anion-exchange buffer A for 30 min, during which the tube was vortexed several times. The buffer was then pipetted off the gel pieces and the process was repeated twice more to extract as much protein as possible from the gel.

### Carotenoid analysis

Extraction and analysis of carotenoids was performed under red light conditions to minimize photo-oxidation and isomerization. Carotenoids were either extracted from gonad tissue with acetone, according to the methods of Symonds *et al*. [12] and Garama *et al*. [23] or from solution by a chloroform:methanol:H_2_O (0.15:0.4:0.3 v/v/v) phase partition method described by Bligh and Dwyer [43]. Carotenoid extracts were evaporated to dryness under oxygen–free N_2_-gas and re-dissolved in 1 mL of methanol.

For analysis by RP-HPLC, carotenoid was extruded through a 13 mm, 0.22 µm Acrodisc GHP membrane syringe-filter (Pall Corporation, NY, USA).

Chromatography was performed according to the methods of Symonds *et al*. [12] and Garama *et al*. [23] using a Gilson 215-liquid handler/injector module coupled to a 321-tandem pump and UV/Vis-156 detector (Gilson incorporated, WI, USA). Carotenoids were separated over a Develosil C30-UG column (Phenomenex Inc. CA, USA) 250 mm × 4.6 mm internal diameter, 5 µm silica bead packing (140 Å pore size) and fitted with a guard-column (Security Guard, Phenomenex). The mobile phase consisted of a gradient of A, methanol with 1% v/v ddH_2_O and 0.01% w/v ammonium acetate and B, tert-butylmethylether (TBME).

The sample was injected onto the column, pre-equilibrated with solvent A, at a flow-rate of 1 mL.min^−1^. Carotenoids were eluted with a gradient of increasing solvent B concentration from 0%–60%, over a volume of 30 mL at a flow-rate of 1 mL.min^−1^. Carotenoids were identified through the comparison of retention times to commercial standards (DHI, Hørsholm, Denmark) and by comparison to the chromatography separation reported in Symonds *et al.* [12].

### Protein identification

Protein eluted and re-concentrated from 1D native-PAGE (see native purification) was analyzed under reducing (5% w/v β-mercaptoethanol) and denaturing conditions (3% w/v SDS) on a 12.5% acrylamide, Tris-glycine pH 8.8, 1D mini-gel (1D SDS-PAGE). The protein-stained band of interest was excised from the gel for analysis by mass spectrometry.

Mass spectrometry for native and recombinant protein identification was performed according to the following procedure. Protein bands from 1D SDS-PAGE were subjected to in-gel digestion with either sequencing grade modified trypsin (Promega, WI, USA) or endoprotienase Glu-C derived from *Staphylococcus aureus* V8 (Sigma-Aldrich, MO, USA), performed using an automated protein digestion robot (DigestPro Msi, Intavis AG, Cologne, Germany), according to the method of Shevchenko *et al.* [44]. Eluted peptides were evaporated to dryness in a Savant Speed Vac SC 100 vacuum concentrator (Thermo Scientific, MA, USA) and re-dissolved in 30% v/v acetonitrile and 0.1% v/v trifluoroacetic acid (TFA). A 1:2 mixture of sample:matrix (10 mg.mL^−1^ α-cyano-4-hydroxycinnamic acid, 10 mM ammonium dihydrogen phosphate, 0.1% v/v TFA and 65% v/v acetonitrile) was applied to a 384 position Opti-tof mass spectrometry sample plate (Applied Biosystems, MA, USA). Analysis was performed on a 4800 MALDI TOF/TOF analyzer (Applied Biosystems), with spectra acquired in positive ion mode and 800 laser pulses per sample spot. The 20 strongest signals were isolated for collision-induced dissociation analysis, which was conducted in 2 kV mode with 4,000 laser pulses per spot. Air, pressurized to 1x^−6^ torr, was used as the collision gas.

Peptide-mass peak data were submitted to the Mascot server (Matrix Science, www.matrixscience.com Accessed 29 December 2012) [45] and an MS/MS ion search was performed. Files were searched against the NCBI-Echinodermata and NCBI-non-redundant databases which contained the *Strongylocentrotus purpuratus* genome [46]. Peptide and mass tolerances of 75 ppm and 0.4 Da respectively were used for the Mascot searches. In addition, up to three missed cleavages were allowed and the variable modifications of oxidized methionine, carbamidomethyl cysteine, pyroglutamate/glutamine were included. The *S. purpuratus* amino acid sequence, identified through MASCOT, was searched against the NCBI-non-redundant database using PSI-BLAST (http://www.ncbi.nlm.nih.gov Accessed 5 January 2013) [47], to identify homologous sequences. Additional peptide matching was performed by Mascot search against a user database containing the *E. chloroticus* EBP (EBP(Ec)) amino acid sequence.

### Sequence alignments

Amino acid sequences for alignments were obtained from the Universal Protein Resource Knowledge Base (Uniprot Kb, www.uniprot.org Accessed 10 July 2013) [48]. Global and local pair-wise sequence alignments were performed using EMBOSS-Needle and EMBOSS-Matcher respectively provided by EMBL-EBI (www.ebi.ac.uk/Tools/psa/. Accessed 15 November 2013) [49]. Multiple sequence alignments were performed using Multiple Sequence Comparison by Log Expectation (MUSCLE), provided by EMBL-EBI (www.ebi.ac.uk/Tools/msa/muscle/. Accessed 2 December 2013) [50], [51]. Shading of alignments was done using T_E_X shade version 1.24 (a L^A^T_E_X package, www.ctan.org. Accessed 4 December 2013) [52]. Thresholds were set to 50% for similar residues and 80% for identity.

### Cloning, expression and purification of apo-rEBP and apo-rEBP-C61S

The EBP cDNA sequence was obtained from the *de novo* assembly of the *E. chloroticus* transcriptome [53]. EBP cDNA was synthesized from *E. chloroticus* gonad mRNA, obtained from Gillard *et al*. [53], using a Transcriptor High Fidelity cDNA Synthesis kit (Roche Applied Science) with forward and reverse primers 5′ CTGATACTCATATGCCTACCGACTTCAGCG 3′ and

5′ GACTCGAGGGGTCGGTTCTGTATATCTTAGAC 3′ respectively.

EBP cDNA was cloned into a pET-28 plasmid (Novagen Merck-Millipore, Darmstadt, Germany), site directed mutagenesis was performed as described by Liu and Naismith [54] using the forward and reverse primers

5′ TGCGCGAAGCAATGAATACAGCTTCGTGGTCGGGTC 3′ and

5′ CATTGCTTCGCGCAGCTGCTTGGCTCTTGATTGTG 3′, respectively.

Plasmid vectors containing either mutant or wild type EBP were transformed into *Escherichia coli* BL21(DE3) (Stratagene, La Jolla, USA), which was confirmed by DNA sequencing performed by Genetic Analysis Services, University of Otago, Dunedin, New Zealand. The cell cultures were grown to OD_600 nm_  =  0.6 and induced with 0.125 mM isopropyl-β-D-1-thiogalactopyranoside (IPTG, Sigma-Aldrich) and transferred to 27°C. The cells were harvested by centrifuging 6 h post-induction. Apo-rEBP and apo-rEBP-C61S were purified according to protocols adapted from Bornhorst and Falke [55] and Tropea *et al*. [56]. Cell pellets were resuspended in buffer containing 20 mM HEPES pH 8.0, 0.1 M NaCl, 20 mM imidazole, 10 mM β-mercaptoethanol, with inclusion of cOmplete mini EDTA-free protease inhibitor tablets. Cell lysis was performed by sonication in an ice-bath, using a Sonifier cell disruptor (Qsonica, CT, USA) set to pulse mode at 40% power out-put. Three, 4 min sonication cycles with a 2 min recovery period between each cycle were performed. Cellular debris was pelleted by centrifuging for 15 minutes at 8,000 *g*, following which the supernatant was decanted and filtered through a 25 mm 0.45 µm PTFE filter.

Filtered lysate was injected onto a 5 mL His-trap Ni^2+^-nitrilotriacetic acid (NTA) column (GE Healthcare), at a flow rate of 5 mL.min^-1^. The column was washed with 25 mL of IMAC buffer A (20 mM 4-(2-hydroxyethyl)-1-piperazineethanesulfonic acid (HEPES) pH 8.0, 20 mM imidazole and 0.5 M sodium chloride) and then bound protein was eluted over a 100 mL gradient from 0-100% buffer B (buffer A and 0.5 M imidazole) and monitored at 280 nm. Fractions containing apo-rEBP were pooled and dialyzed overnight at 4°C against IMAC buffer A, which contained Tobacco Etch Virus Protease (TEVpro) at a TEVpro:sample ratio of 1∶30 w/w.

The dialyzed sample was re-passed through the His-Trap Ni^2+-^NTA and the column effluent was collected and spin-filter concentrated to a volume of 5 mL. The concentrated sample was filtered through a 0.22 µm syringe filter and injected onto a HiPrep 16/60 Sephacryl S-100 HR gel-permeation column (GE Healthcare), pre-equilibrated with 50 mM HEPES pH 8.0, 0.1 M NaCl and 10% v/v glycerol. Proteins were eluted with 120 mL isocratic flow at 0.5 mL.min^-1^.

### Secondary structure prediction

*Ab initio* secondary structure predictions for the EBP amino acid sequence were made using five different web servers YASPIN [57] (www.ibi.vu.nl/programs/yaspinwww/Accessed 21 August 2013), SPINEX [58] (http://sparks.informatics.iupui.edu/SPINE-X/. Accessed 21 August 2013), NetSurfP [59] (http://genome.cbs.dtu.dk/services/NetSurfP/. Accessed 21 August 2013), PSIPRED [60] (http://bioinf.cs.ucl.ac.uk/psipred/. Accessed 21 August 2013) and PHD [61] (www.predictprotein.org/. Accessed 21 August 2013). The proportions of helix, strand and coil were calculated for each program and the mean of all five predictions produced a consensus assignment for each amino acid.

Protein samples for circular dichroism spectroscopy (CD) were dialyzed overnight at 4°C against 5 mM NaH_2_PO_4_/Na_2_HPO_4_, pH 8.0 and then the concentration of the protein sample was adjusted to 0.3 mg.mL^-1^. All measurements were conducted at 18°C in a 0.2 mL cylindrical CD quartz cuvette with a 0.1 cm path-length, using an Olis CD module (On-Line Instrument Systems Inc. CA, USA) with oxygen-free N_2_-gas flow through the lamp, monochromator and sample chamber at 10, 25 and 10 L.min^-1^ respectively. Elipticity data were collected between 260 nm and 190 nm with a 2 nm bandwidth and integration time set as a function of high voltage. Five scans were collected per sample with the calculated mean reported as mean residue elipticity [θ]_MRE_ (deg.cm^2^ dmol^-1^) [62].

Protein secondary structure was predicted from the CD data using the CDPro software package (http://lamar.colostate.edu/~sreeram/CDPro/main.html. Accessed 5 September 2013) [63], which included the algorithms CDSSTR [64], [65], CONTINLL [66], [67] and SELCON 3 [64], [68], [69]. Structure prediction was performed using SMP56, SDP48 and SP43 reference sets for each algorithm, final predictions for apo-rEBP and apo-rEBP-C61S are reported as an average across each reference set and algorithm combination.

### Reconstitution of echinenone with apo-rEBP and apo-rEBP-C61S

Echinenone used for reconstitution experiments was extracted from *E. chloroticus* gonad tissue and purified by RP-HPLC, as previously described. A 0.5 mL aliquot of carotenoid extract was injected on to a C30-UG RP-HPLC column and fractions were collected between 25 and 30 min. Fraction volume was set to 200 µL within peaks and 1.5 mL for non-peaks, with a A_445nm_ peak detection limit of 40 mV. Fractions from the central portion of the 9′-*cis*-echinenone peak were pooled across multiple RP-HPLC runs and the solvent was evaporated under oxygen-free N_2_-gas. All traces of moisture were removed by overnight desiccation in a Savant Speed Vac vacuum concentrator. Powdered echinenone was dissolved in 1 mL methanol and the purity of 9′-*cis*-echinenone was evaluated by RP-HPLC, to verify that there was <10% all-*trans*-echinenone in the sample.

Incorporation of echinenone into recombinant protein was based on a method described by Rao *et al*. [26]. The concentrations of purified apo-rEBP and apo-rEBP-C61S were determined by A_280nm_ measurements and then adjusted to 2 mg.mL^-1^. Bovine serum albumin (BSA, NZ sourced from Invitrogen, Auckland, NZ) at a concentration of 2 mg.mL^-1^ was used as a positive control. A 0.5 mL aliquot of each protein was incubated with 10 nmol and 100 nmol of RP-HPLC purified 9′-*cis*-echinenone for 1 h at 37°C, with rotation. Samples were then dialyzed overnight in 10 kDa MWCO tubing against S-100 gel-permeation buffer at 4°C and then centrifuged at 10,000 *g* for 10 min with retention of the clarified supernatant.

The reconstituted holo-rEBP-C61S was passed through a 16/60 S-100 gel permeation chromatography column (GE Healthcare), to remove any remaining unbound carotenoid and then reconcentrated to 1 mg.mL^−1^. The absorbance spectrum was then measured between 260 nm and 700 nm in a Cary 100 UV-Vis spectrophotometer (Aligent Technologies, CA, USA), with gel-permeation chromatography buffer (20 mM HEPES, pH 8.0) as the reference solution. Spectra were also obtained for apo-rEBP-C61S (diluted with buffer to 1 mg.mL^−1^) and purified 9′-*cis*-echinenone (0.9 mg.mL^−1^ in methanol, which was also used as the reference solution for 9′-*cis*-echinenone).

## Results and Discussion

### Partial purification of a carotenoid-protein complex from *E. chloroticus* gonads

A CBP complex was extracted and purified from the gonad homogenate of the sea urchin *E. chloroticus* and represents a novel addition to the protein class. The approach to purification was to successively fractionate the homogenate and at each step select for fractions that exhibited absorbance at both 280 nm and 454 nm, indicative of the presence of protein and carotenoids respectively.

Due to the high lipid content of sea urchin gonad tissue, which comprises approximately 28% of the dry gonad tissue weight [70], the homogenate required extensive pre-processing steps prior to chromatography.

**Figure 1:**
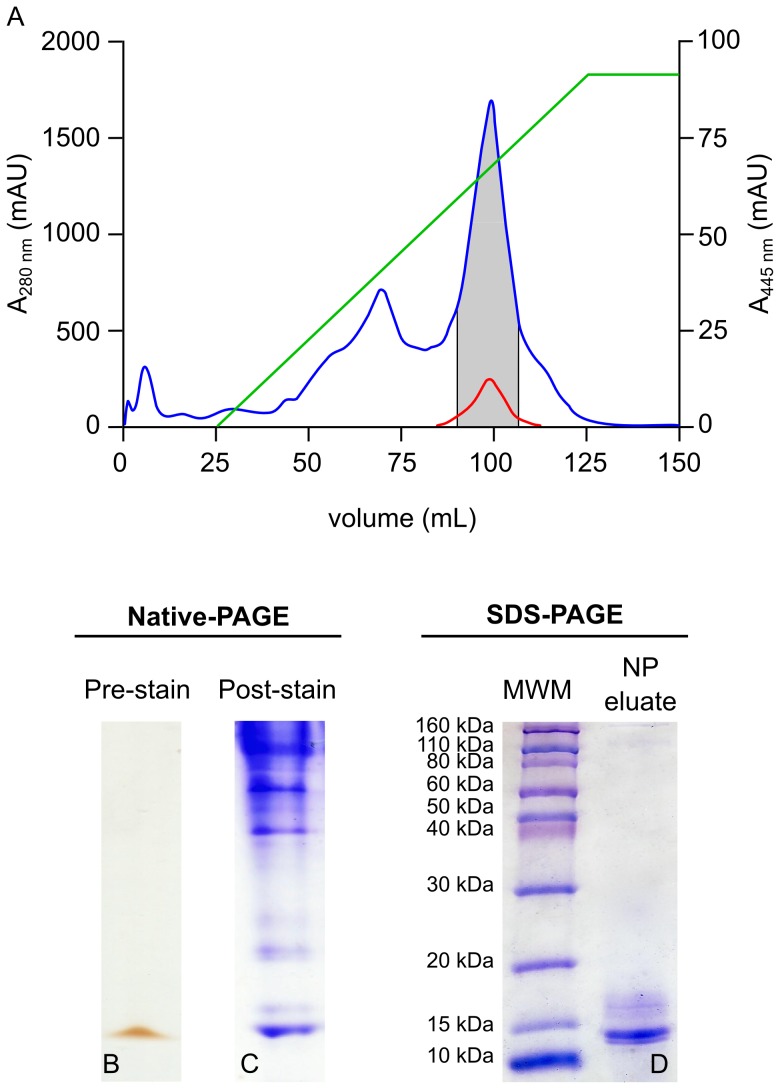
Isolation of a putative CBP by 1D native-PAGE. *E. chloroticus* gonad-soluble protein extract was fractionated by anion exchange chromatography, **A**. Gonad protein extract corresponding to 1 g wet weight gonad was loaded onto a 5 mL HiTrap Q-Sepharose column and bound protein was eluted by a 0–100% gradient of 1M NaCl (green line). The absorbance of the column effluent was monitored at 280 nm (blue) and 445 (red). Fractions absorbing at both 280 nm and 445 nm (grey zone) were pooled concentrated. **B**. A 20 µL aliquot of the concentrate was analyzed on 1D native-PAGE, shown prior to staining. **C**. A duplicate loading on 1D native-PAGE was stained with Coomassie blue R-250. **D**. The yellow/orange band visible on the pre-stained gel in **B**. was excised and the protein was eluted and then analyzed by 1D SDS-PAGE and stained with Coomassie blue R-250.

Removal of lipid also appeared to result in loss of some carotenoids, due to the association of carotenoids with lipoprotein-like complexes [71]. The lipid-depleted CBP-containing sample was fractionated by Q-Sepharose anion exchange chromatography (Figure 1.A) and single–peak fractions exhibiting absorbance at both 280 nm and 454 nm were pooled.

The re-concentrated Q-Sepharose peak was yellow/orange in color, indicating the presence of chromophore. An aliquot of the material was analyzed by 1D native-PAGE. It was hypothesized that under the non-reducing and non-denaturing conditions of 1D native-PAGE the interaction between the chromophore and protein would be preserved. Following 1D native-PAGE a yellow/orange band was observed, which was equivalent in color to the amount of sample applied to the gel (Figure 1.B). Staining the gel with Coomassie blue resulted in the yellow/orange band staining blue (Figure 1.C), indicating the presence of protein. Elution of the yellow/orange colored band from the native PAGE followed by analysis by 1D SDS-PAGE and staining with Coomassie blue displayed a prominent protein band with an apparent molecular weight of between 10 and 15 kDa. Other faintly stained contaminant protein bands were also visible between 15 kDa and 20 kDa (Figure 1.D).

### Identification of an echinenone-binding protein (EBP)

**Figure 2:**
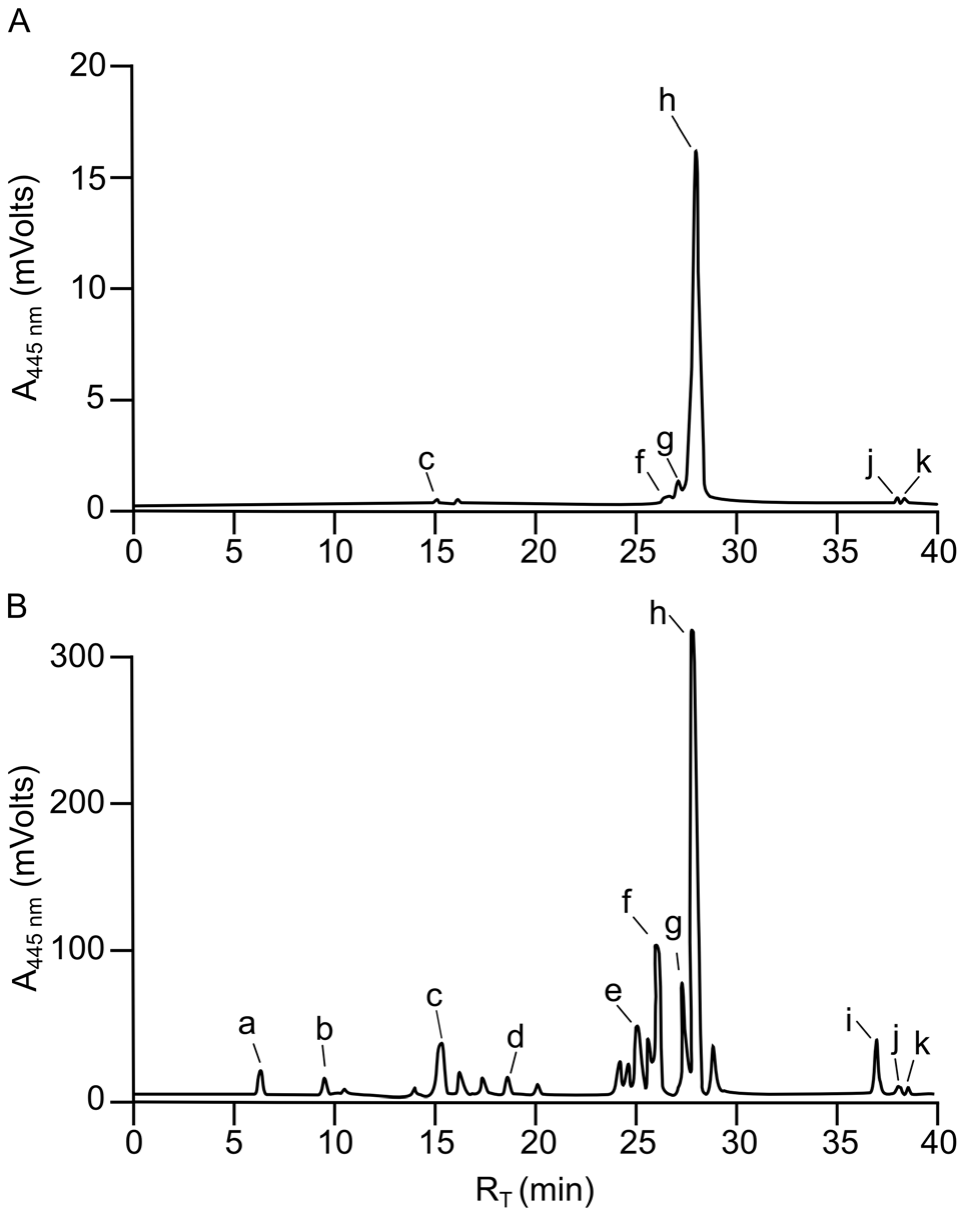
RP-HPLC of carotenoids extracted from EBP and from *E. chloroticus* gonad tissue. Carotenoids extracted from native-PAGE eluate (Figure 1) **A** and from lyophilized whole gonad tissue **B**, were separated by RP-HPLC. Each carotenoid extraction corresponded to a whole gonad, of the same weight, taken from a single animal. For RP-HPLC analysis a 100 µL aliquot of carotenoid was injected onto a C_30_ RP-HPLC and carotenoids were eluted with a methanol/TBME gradient. Column effluent was monitored at 445 nm and carotenoids were identified by comparison of retention times (R_T_) to commercial standards. The carotenoids identified were a. fucoxanthinol, b. fucoxanthin, c. astaxanthin, d. canthaxanthin, e. lutein, f. isozeaxanthin, g. all-*trans*-echinenone, h. 9′-*cis*-echinenone, i. α-carotene, j. all-*trans*-β-carotene and k. 9′-*cis*-β-carotene.

The yellow/orange chromophore was extracted from the eluted native PAGE protein, analyzed by C30 RP-HPLC and was found to consist almost entirely of the carotenoid 9′-*cis*-echinenone. Trace amounts of other carotenoids, including astaxanthin, isozeaxanthin, all-*trans*-echinenone, 9′-*cis*-β-carotene and all-*trans*-β-carotene were also detected (Figure 2.A). The yellow/orange chromophore-protein complex was therefore named echinenone-binding protein (EBP), for the primary ligand associated with the complex when extracted from the native source of sea urchin gonad tissue.

Detection of multiple carotenoid species apparently present with the EBP raised the question of the binding specificity of the EBP. Identification of 9′-*cis*-echinenone as the primary carotenoid associated with the EBP does not necessarily indicate binding preference, relative to other carotenoids. The ligand composition may merely be a reflection of the high concentration of 9′-*cis*-echinenone in the sea urchin gonad (Figure 2.B) [12], [24], with carotenoid incorporation into complexes in proportion to concentrations present in the tissue. However, if that were the case, it would be expected that the composition of carotenoids extracted from EBP would be proportional to that of total carotenoids present in extracts of *E. chloroticus* gonad.

RP-HPLC analysis of total gonad carotenoid extract from the same animal, resolved up to 20 different carotenoids, 11 of which could be identified in comparison with carotenoid standards (Figure 2B). Consistent with previous studies on sea urchin gonad carotenoids, 9′-*cis*-echinenone was the major carotenoid identified, in addition to small amounts of all-*trans*-echinenone, isozeaxanthin, lutein, astaxanthin and α-carotene [12], [24]. The carotenoid profiles of EBP extract and total gonad extract were observed to differ significantly with respect to the carotenoid composition and relative proportions. The ratio of 9′-*cis* to all-*trans*-echinenone in the total gonad carotenoid extract was found to be approximately 3∶1. This ratio was not consistent with the carotenoid extracted from EBP, for which the ratio was approximately 11∶1. In addition, other major gonad carotenoids, lutein and α-carotene, were not detectable in the EBP carotenoid extract. Therefore EBP appeared to exhibit a degree of binding specificity for 9′-*cis*-echinenone but may also be able to bind other carotenoids.

Previous reports of calycin family CBPs have indicated a tendency towards ligand binding promiscuity. Glutathione-S-transferaseP1 from the human macula was co-purified with zeaxanthin but was also able to bind lutein with a similar affinity [27] and carotenoid extracts from a lutein-binding protein, purified from *Bombyx mori*, were found to contain 90% lutein with the remainder consisting of α-carotene and β-carotene [72]. Carotenoids are derivatives of polyisoprenoids and although over 700 have been identified in nature [73], many exhibit very similar structures. Commonly both ends of the carbon chain are cyclized to form ionone rings and diversity is achieved through differences in a single atom or bond position. Therefore, considering the general similarity of carotenoid size and shape, in addition to the miscellaneous groups of ligands reported to be able to be accommodated by the large binding pocket of calycin proteins [34], [74], ligand promiscuity amongst CBPs is not surprising.

The identification of a protein-carotenoid complex, which appears to bind mainly 9′-cis-echinenone, the major gonad carotenoid, has implications for contribution to gonad color. The low abundance of the protein in the gonad suggests that the protein is not likely to be involved in maintaining the carotenoid in a stored state within the gonad. It may be involved in the transport of carotenoid from the viscera into the gonad, or trafficking within the gonad tissue leading to deposition in lipid complexes in the gonad.

### Protein characterization by mass spectrometry

The protein stained band suspected to be the EBP apo-protein was excised from a 1D SDS-PAGE gel (Figure 1.D) for in-gel tryptic digestion and analysis by MALDI TOF/TOF mass spectrometry. The peptide mass data were searched using the Mascot server, against both the NCBI non-redundant and Echinodermata databases, which contain sequences from *S. purpuratus* [46], a Northern hemisphere sea urchin. The peptide mass search resulted in the identification of a hypothetical protein (accession number gi39036640) of 168 amino acids in length (491 base pairs) with an estimated molecular weight of 18.69 kDa (Table 1).

**Table 1:** Identification of EBP by mass spectrometry.

Identity	Accession	Mass (Da)	Peptide	Score^a^
Predicted; uncharacterised protein, LOC762462 from S. purpuratus	Gi:390363640	18688	EIIDGQMVTTVSK	63

a Scores > 56 indicate a significant match (p<0.05).

The hypothetical status of the matched sequence indicated that although it had not been annotated in the *S. purpuratus* database, it corresponded to a predicted open reading frame. The release of the *S. purpuratus* genome sequence in 2006, represented the first whole genome sequence of a motile, free-living marine invertebrate [46]. Gene prediction software estimated the number of genes at approximately 23,300 and a subsequent in-depth analysis by the sea urchin research community resulted in the functional annotation of approximately 9,700 (42%) of the identified genes [75]. Furthermore, the recent publication of an *S. purpuratus* transcriptome identified transcripts for the majority of the predicted genes, approximately 21,000, including gi39036640 (http://www.spbase.org/SpBase/wwwblast/blast-run.php. Accessed 5 May 2014) [76]. However, since the publication of the genome sequence no significant advancements have been reported toward functional annotation, leaving a large proportion of the genome assigned as predicted hypothetical gene sequences.

Multiple attempts to characterize EBP by mass spectrometry resulted in repeated matches to the *S. purpuratus* gi39036640 sequence. However, this identification was consistently the result of a single, significant (p<0.05) peptide match (Table 1). Only identical peptides, of minimally 5 amino acids, will result in a match between two sequences using the Mascot program [45]. Therefore the absence of other peptide matches may be due to insufficient sequence identity between *E. chloroticus* and *S. purpuratus* at this locus. However, the single peptide identified was sufficient to obtain the *E. chloroticus* EBP (EBP(Ec)) cDNA sequence, through a BLASTx search against a recently reported *E. chloroticus* transcriptome assembly [53]. The mass spectrometry peptide mass data was then searched against the EBP(Ec) cDNA sequence, which resulted in an additional 10 peptide matches (Table S1) and 66% sequence coverage (Figure S1).

The EBP(Ec) open reading frame (ORF) cDNA sequence was predicted to be 396 base pairs in length, resulting in a translated sequence of 131 amino acids (Figure S2). The molecular weight of the protein product was calculated to be 14.45 kDa from the amino acid sequence, which was significantly smaller than the predicted ORF of EBP *S. purpuratus* homologue, at 18.69 kDa. Therefore the possibility was considered that only a partial EBP(Ec) sequence had been identified and the sequence upstream of the ATG was analyzed for the presence of an alternative start codon. However, none were identified, although an in-frame up-stream terminator codon, at -72 bp (Figure S2), limited the search to that point. The completeness of the *E. chloroticus* transcriptome assembly was validated and an average of 8.2-fold sequence coverage was established [51], therefore the EBP(Ec) sequence prediction is likely to be correct. In addition, a molecular weight of 14.45 kDa for EBP(Ec) is consistent with the protein stained band, which had an apparent mass of between 10 and 15 kDa on 1D SDS-PAGE (Figure 1.D).

**Figure 3:**
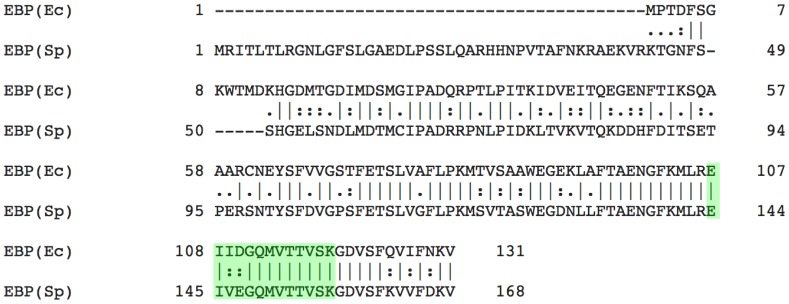
Global pairwise alignment of EBP(Ec) and EBP(Sp) amino acid sequences. A global pairwise alignment of EBP(Sp) and EBP(Ec) amino acid sequences was performed using EMBOSS Needle. The peptide sequence derived by mass spectrometry of an in-gel digest of EBP(Ec) is highlighted in green.

A global pairwise sequence alignment of EBP(Ec) and EBP(Sp) indicated a 45% sequence homology between the two species, with greater homology exhibited towards the C-termini (Figure 3). The molecular weight difference between the two protein products was also made apparent; a large (41 amino acid) N-terminal truncation of EBP(Ec) with respect to EBP(Sp) (Figure 3). In addition the alignment highlighted a curiosity. Location of the mass spectrometry peptide matched to the *S. purpuratus* sequence by Mascot search (highlighted in Figure 3), indicated a 2 amino acid mismatch between the two species. The sequence of the 13 amino acid peptide from *S. purpuratus* was EIVEGQMVTTVSK (Table 1), however in the *E. chloroticus* peptide sequence the 3rd and 4th amino acids V and E are substituted for I and D, respectively. The change from V to I resulted in a +14.0 Da mass difference but conversely the change from E to D resulted in a −14.0 Da mass difference. Therefore, although the peptide sequences were not identical, the two substitutions resulted in compensatory mass changes and hence identical peptide masses that resulted in peptide identification by Mascot.

### Recombinant production of EBP

Attempts to purify the EBP complex in *E. chloroticus* gonad tissue resulted in low protein yield, which limited further characterization experiments. In addition, the EBP complex was unable to be obtained highly purified from the gonad tissue, with several minor contaminant species <20 kDa apparent on 1D SDS-PAGE (Figure 1.D). However, the identification of the EBP cDNA sequence from the *E. chloroticus* transcriptome [53] enabled EBP to be produced recombinantly (apo-rEBP). Furthermore, recombinant protein production also overcame factors such as the high lipid content of sea urchin gonads [70], which was problematic when purifying EBP from its native source.

**Figure 4:**
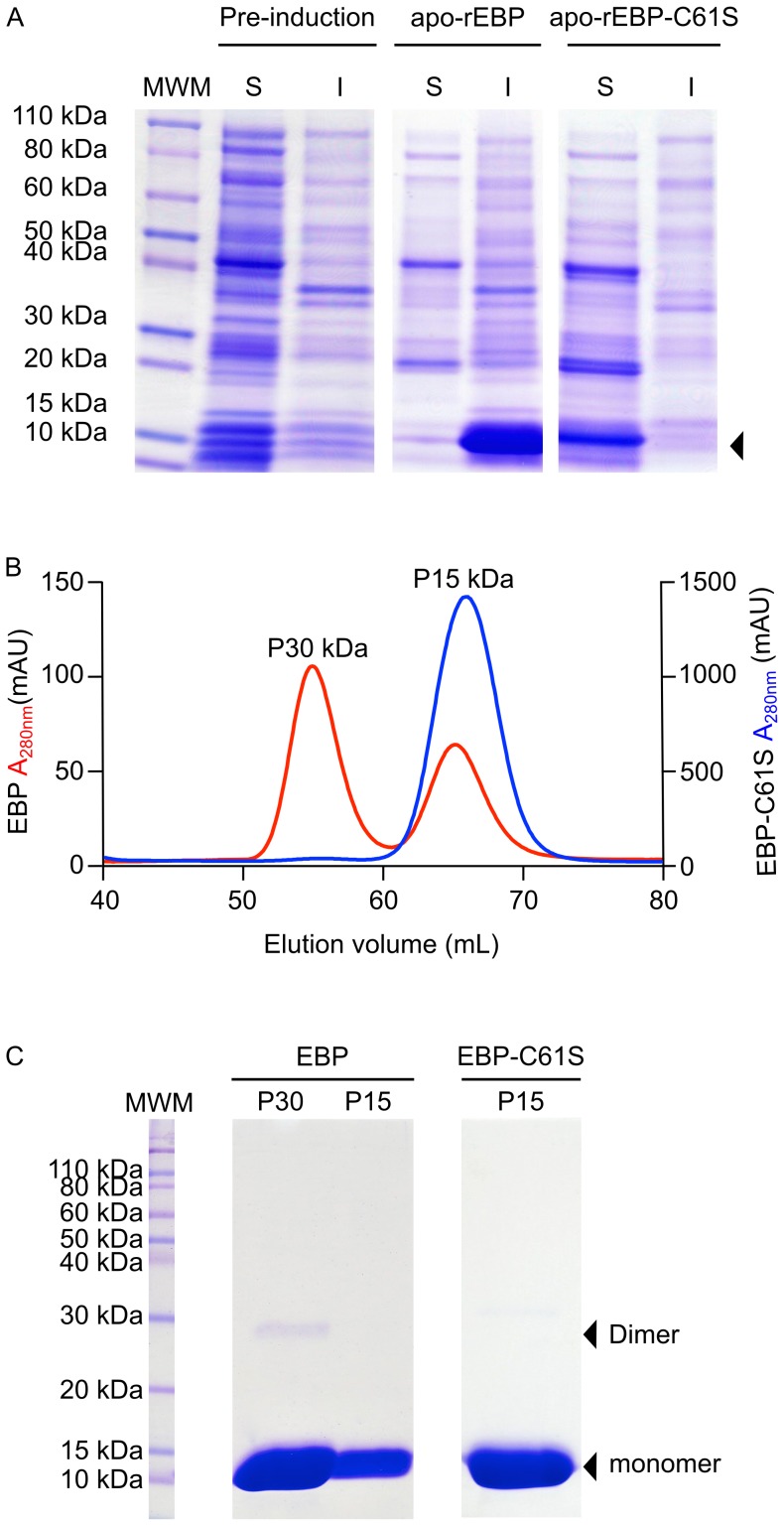
Expression and purification of recombinant apo-EBP and apo-EBP-C61S. Apo-rEBP and apo-rEBP-C61S were produced using *E.coli* expression strain *BL21(DE3)*, Expression levels were evaluated on reducing 1D SDS-PAGE, prior to induction and 6 h post-induction, **A**. Soluble (S) and insoluble (I) fractions of bacterial cell lysate are indicated. Arrowhead indicates the position of EBP and EBP-C61S bands, the identities of which were confirmed by mass spectrometry (Figure S1). Apo-rEBP and apo-rEBP-C61S were purified from the soluble fractions of the bacterial cell lysates. Gel-permeation chromatography was performed under non-reducing conditions for the final stage of purification on a 16/60 Sephacryl S-100 column, with the effluent monitored at 280 nm, **B**. Apo-rEBP is shown on the left *y*-axis (red) and apo-rEBP-C61S on the right *y*-axis (blue). The molecular weight of the eluting species was estimated with reference to the manufacturers Sephacryl S-100 column standard curve. The fractions within each peak were pooled and a 10 µL aliquot of each was analyzed on reducing 1D SDS-PAGE, **C**. The positions of the monomeric and dimeric forms of protein are indicated by black arrowheads.

The expression of apo-rEBP was analyzed by 1D SDS-PAGE (Figure 4.A). Following induction of *BL21(DE3) E. coli* liquid cultures with IPTG, a prominent protein stained band, that was not present prior to induction, was observed to migrate at an apparent mass of between 10 and15 kDa (Figure 4.A arrow). The appearance of the protein in the expected size range enabled putative identification as apo-rEBP, which was confirmed by MALDI TOF/TOF mass spectrometry (Figure S1 and Table S1). The large protein-stained bands on 1D SDS-PAGE, corresponding to apo-rEBP, indicated that the protein was over-expressed in significant quantity. However, approximately 80% of the protein appeared to be insoluble upon cell lysis (Figure 4.A) and it was likely that the majority of the protein was present in inclusion bodies.

Recombinant production of apo-proteins may result in solubility issues as the ligand helps to stabilize the protein in the correct conformation, particularly if the ligand is hydrophobic [77]. As carotenoids are highly hydrophobic, the EBP would be expected to have a reasonable proportion of non-polar amino acids for interacting with the carotenoid ligand. These residues are usually buried, but in the absence of the ligand may become solvent exposed, resulting in hydrophobic patches on the surface of the protein causing aggregation. However, numerous lipocalins and FABPs have been successfully expressed as apo-proteins [29], [72], [78], [79], [80]. Although various conditions for the expression of apo-rEBP were trialed no improvements upon the initial proportion of soluble protein could be achieved.

Reduced solubility amongst recombinant proteins may be due to incorrect disulphide bond formation [77], [81]. The EBP(Ec) amino acid sequence contains a single cysteine residue at position 61 (Figure S2), therefore no intramolecular disulphide bonds were possible. However, the formation of cross-links between the cysteines of two separate apo-rEBP molecules was a possibility [82]. Strain placed upon the dimer through the formation of a non-nascent disulphide bond, may result in conformational changes that expose hydrophobic amino acids and promote aggregation at the high protein concentrations produced by recombinant over-expression. Although reducing agent was included in the lysis buffer, it appeared that this was not effecitve for the recovery of soluble protein from what was likely inclusion body aggregates. To test the hypothesis that inter-chain disulfide formation contributed to the low solubility of apo-rEBP, site directed mutagenesis was used to replace the cysteine at position 61. The pairwise sequence alignment of EBP(Ec) and EBP(Sp) indicated that the cys61 was not conserved between the two sea urchin species, with a serine present at the corresponding position of EBP(Sp) (Figure 3). This suggested that mutation of the cysteine to serine might result in minimal perturbation to protein structure and function.

Expression of the recombinant EBP cysteine to serine mutant (apo-rEBP-C61S) produced a protein that on 1D SDS-PAGE migrated with apparent mass of between 10 and 15 kDa (Figure 4.A). The C61S mutation was confirmed by DNA sequencing the mutant in the plasmid construct (Figure S3) and by MALDI TOF/TOF mass spectrometry of the protein product (Figure S1 and Table S1). In contrast to apo-rEBP, apo-rEBP-C61S was recovered exclusively from the soluble fraction of the *E. coli* cell lysate, with none detected in the insoluble fraction on 1D SDS-PAGE (Figure 4.A). Despite the low solubility of apo-rEBP, both mutant and wild type proteins were purified in order to assess the effects of the mutation on protein structure and function.

Gel-permeation chromatography was conducted under non-reducing conditions as the final step in the purification of each protein. Two peaks were observed on the chromatogram of apo-rEBP, corresponding to the expected molecular weights of monomeric and dimeric forms of the protein (Figure 4.B). 1D SDS-PAGE supported the dimer hypothesis; in addition to the protein band migrating at an apparent mass of between 10 and 15 kDa, a faint band was observed migrating at approximately 30 kDa (Figure 4.C), likely the result of incomplete reduction of disulphide bonds prior to PAGE. The apo-rEBP dimer is not likely to be a physiologically important state, as no evidence of dimerization was observed during the native purification of the holo-protein. Instead dimerization appears to occur as result of high concentrations of the apo-protein. In the holo-protein complex C61 may be interacting with the ligand, or otherwise shielded from adjacent proteins. In contrast a single peak, corresponding to the monomeric protein was observed on the gel-permeation chromatogram of apo-rEBP-C61S. In addition, no other protein-stained bands were visible on 1-D SDS-PAGE, indicating the purity of apo-rEBP-C61S.

### Apo-rEBP-C61S binds echinenone *in vitro*

RP-HPLC-purified 9′-*cis*-echinenone was incubated with apo-rEBP and apo-rEBP-C61S in an attempt to effect ligand binding. Reconstitution of protein and carotenoid was visually assessed by the intensity of the yellow coloring following the removal of unbound carotenoid. Apo-rEBP appeared to retain very little color even at the highest concentration of 100 nmol 9′-*cis*-echinenone (Figure S4). However the EBP-C61S solution was light yellow and proportional in color to the BSA positive control (Figure S4), which was included as albumins are known to bind and transport carotenoids in the serum [83].

The C61S mutation did not appear to inhibit the ability of apo-rEBP-C61S to bind the carotenoid ligand. However, the apparent inability of apo-rEBP to bind 9′-cis-echinenone may be due to the observed tendency of the protein to dimerize and aggregate, which may result in changes to the protein structure or obscured access to the ligand-binding pocket.

**Figure 5:**
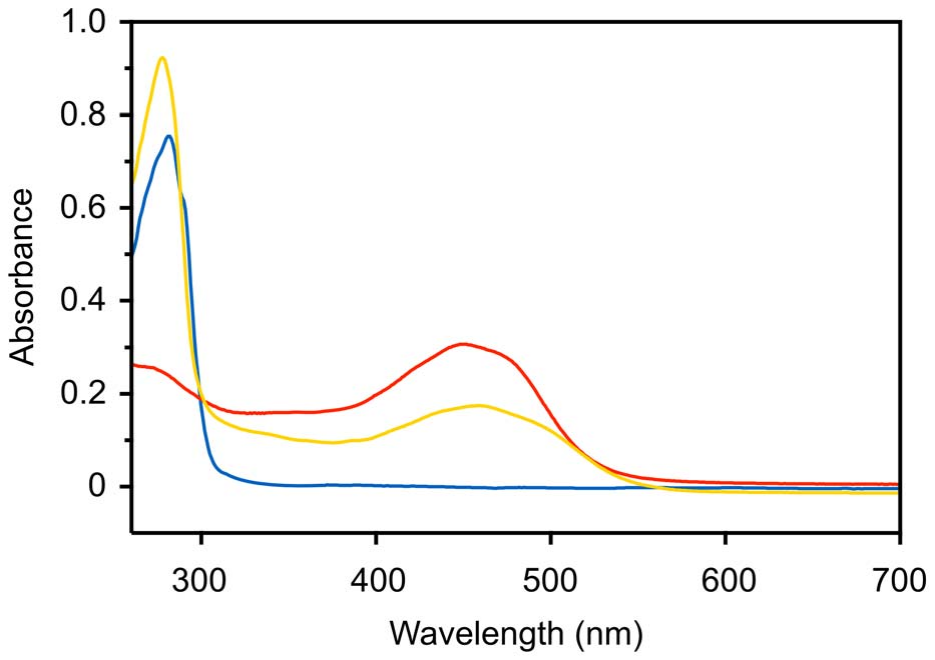
Absorbance spectra of apo and holo-rEBP-C61S. Following protein-ligand reconstitution and removal of unbound carotenoid the absorbance spectrum of holo-rEBP-C61S (1 mg.mL^-1^) was measured between 260 nm and 700 nm, against a reference solution of 20 mM HEPES pH 8.0 (yellow line). Spectra of 1 mg.mL^-1^ apo-rEBP-C61S in 20 mM HEPES pH 8.0 (blue line) and purified 9′-*cis*-echinenone at 0.9 mg.mL^-1^ in methanol (red line) are shown for comparison.

The formation of holo-rEBP-C61S was confirmed by measuring the absorbance spectrum between 260 and 700 nm. Prior to reconstitution, apo-rEBP-C61S exhibited a single peak at 280 nm corresponding to aromatic amino acid side chains (Figure 5), whereas purified 9′-*cis*-echinenone exhibited the characteristic absorbance spectrum of carotenoids. A broad two-shouldered peak was observed between 400 nm and 500 nm, which is due to an electronic transition to the first excited state affected by low energy visible wavelengths of light [73], [84]. The broad peak observed below 300 nm is not due to the presence of protein but is most likely a *cis*-peak, which occurs due to the decreased symmetry of *cis*-geometric isomers, relative to all-*trans* isomers [84]. The decrease in molecular symmetry lowers the energy required to effect the usually forbidden electronic transition from the 1A_g_ orbital to that of 2A_g_ [84]. Both protein and carotenoid elements were observed in the spectrum of holo-rEBP-C61S, indicating successful reconstitution of protein and ligand. However the *cis*-peak was obscured by absorbance at 280 nm, although an additive effect may account for a small increase in peak height relative to apo-rEBP-C61S.

The absorbance spectra of 9′-cis-echinenone and holo-rEBP-C61S indicated an approximately 8 nm bathochromic shift (red-shift) upon ligand binding. In methanol the carotenoid peak of purified 9′-*cis*-echinenone was centered at 450 nm with shoulders at 429 and 466 nm, which were observed to be red-shifted to 458, 437 and 475 nm in holo-rEBP-C61S. The observed spectral shift is consistent with reports of other CBPs for which small bathochromic shifts of between 4 and 38 nm are most common [29], [39], [85], [86].

### EBP is a member of the FABP family

An NCBI-BLASTp search [47] of the EBP(Ec) amino acid sequence was performed to obtain more information about the previously uncharacterized protein. The conserved domain function of BLASTp predicted that the protein belonged to either the lipocalin or FABP protein families. As expected EBP(Sp) was identified as the most closely related sequence. However the next highest scoring alignments, with sequence identities of 41-35% corresponded to intestinal and liver FABP-like proteins, from *S. purpuratus* and intestinal FABP FABPI) from *Bos taurus* (Table 2).

**Table 2:** Top five significant results from BLASTP search of EBP(Ec) homology.

Identification	Organism	Accession no.	E-value	% Identity
Predicted Uncharacterised protein	*S. purpuratus*	XP_00119808.2 (GI:390363640)	2e-51	65%
Predicted FABP intestinal-like	*S. purpuratus*	XP_789999.1	1e-25	41%
Predicted FABP-2 liver-like	*S. purpuratus*	XP_782066.1	7e-22	35%
Predicted FABP liver-like	*S. purpuratus*	XP_785180.2	2e-10	35%
FABP Intestinal	*Bos taurus*	NP_001020503.1	1e-9	34%

A multiple sequence alignment (MSA) was performed in order to further explore the relationship of EBP(Ec) and EBP(Sp) to members of the FABP family. Sequences used in the alignment represented several different sub-classes of FABP including: intestinal type (FABP2), liver type (FABP1) and adipocyte lipid-binding protein (ALBP) from *Rattus norvegicus* and retinol-binding protein (RBP) and cellular retinoic acid-binding protein (cRABP) from *Homo sapiens*.

**Figure 6:**
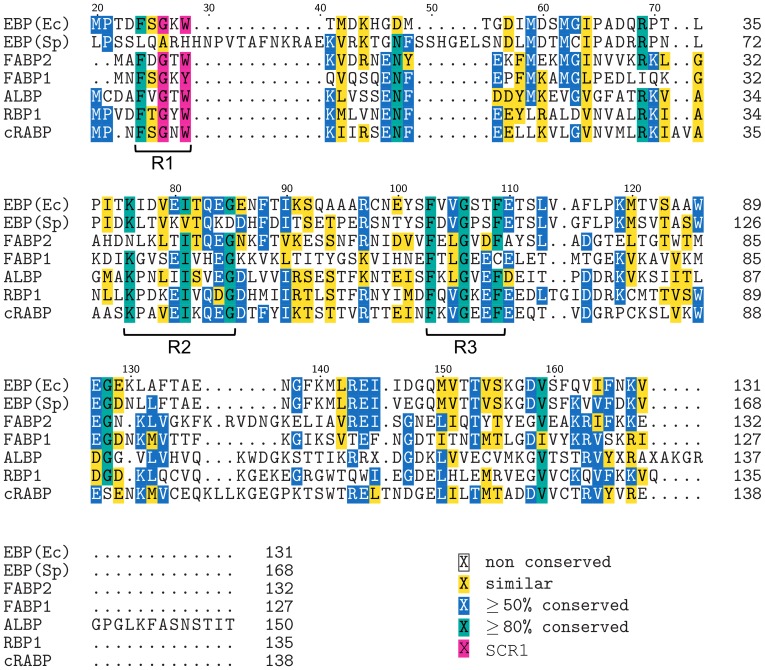
Multiple sequence alignment of EBP(Ec) and EBP(Sp) with members of the FABP family. A multiple sequence alignment of EBP(Ec) and EBP(Sp) with five members of the FABP family was performed using MUSCLE. The five FABP sequences used in the alignment were; intestinal-FABP-2 (FABP2), liver-FABP-1 (FABP1), adipocyte lipid-binding protein (ALBP) from *Rattus norvegicus*, retinol-binding protein-1 (RBP1) and cellular retinoic acid-binding protein (cRABP) from *Homo sapiens*. Positions where ≥50% of residues are similar are shaded yellow. Positions where residue identity is ≥80% are shaded blue. Positions with ≥80% residue identity are shaded green. SCR1 (≥80% identity) is shaded magenta and the three regions of clustered ≥80% amino acid identity are labelled R1, R2 and R3. Amino acid position numbering is with respect to EBP(Sp), for which the first 20 amino acids are not shown.

From the MSA it was apparent that at 168 amino acids, EBP(Sp) is considerably larger in size than not only EBP(Ec) but the other FABPs analyzed as well (Figure 6). ALBP is the next largest FABP at 150 amino acids, however the other proteins, including EBP(Ec), clustered within a uniform size rage of 127 to 138 amino acids, which is typical of FABP [87]. The larger size of EBP(Sp) appears primarily to be due to three short N-terminal proximal insertions of 19, 12 and 5 amino acids, relative to the other FABP sequences analyzed (Figure 6).

The MSA also indicated a number of highly conserved amino acids (≥80% conserved), which generally appear to cluster into 3 regions (R1, R2 and R3; Figure 6). The first region includes the –GXW- (where X is any amino acid) sequence motif (Figure 6, magenta shading), which is conserved within the calycin protein superfamily [88]. The motif is situated within a greater region of structural similarity, known as structurally conserved region 1 (SCR1), which encompasses an N-terminal short 3_10_-like helix and β-strand A [34], [35], [88]. However, other than the three regions with clusters of >80% conserved amino acids, the overall sequence similarity between the proteins was low. Sequence homology amongst members of the FABP family can vary from 70% to as low as 20% [87], and the relationship of EBP(Ec) to sequences analyzed was consistent with the lower end of this range. Pairwise local alignments between EBP(Ec) and the FABPs indicated sequence homology between 29.8% and 21.1% (Table S2), however this is reflective of a comparison between evolutionarily distant species such as invertebrate and mammalian sequences.

FABP are known as intracellular lipid transporters, however they have also been reported to bind an array of other small hydrophobic molecules [37], [38], [87]. The ellipsoidal binding cavity of FABPs is well suited for elongated molecules such as fatty acids and retinoids. Although C_40_ ionone-ring carotenoids are larger than the 14-16 carbon fatty acids, the FABP calyx provides a larger volume than required and is known to accommodate larger, bulkier ligands [37], [82]. Fatty acids are orientated with the carboxylate group buried deep within the calyx [87] and stabilized by interactions with proximal arginine side chains [89]. An analogous mode of binding can be envisaged for 9′-*cis*-echinenone, with the mono-keto group of the xanthophyll, buried within the pocket. Identification of EBP(Ec) as an intracellular transport protein is also consistent with the low yields observed when purifying the complex from the native source. In tissues containing high proportions of lipid FABPs are reported to only contribute 1–5% of the soluble cytosolic protein [90].

**Figure 7:**
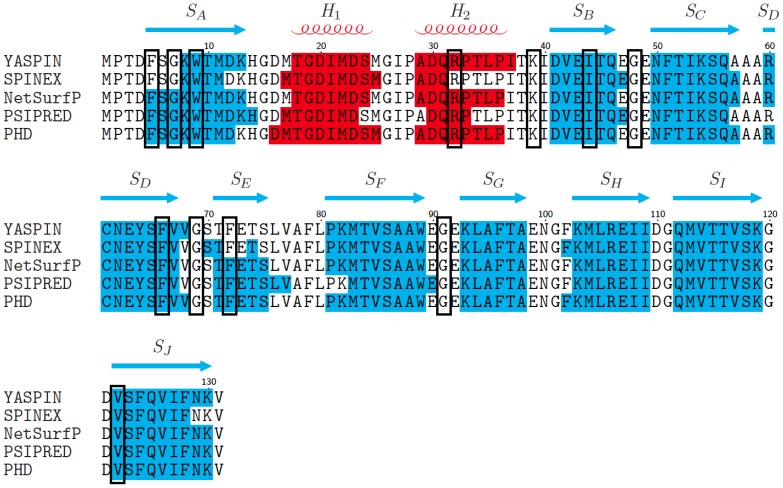
*Ab initio* secondary structure prediction of EBP. Comparison of secondary structure predictions for EBP from five different web-based servers; YASPIN, SPINEX, NetSurfP, PSIPRED and PHD. Blue shaded regions indicate β-strand structures, red shaded regions helical structures and unshaded regions, coils. The consensus prediction, defined as the prediction made by 3/5 or more of the servers, is displayed above the sequence. Blue arrows represent β-strand structures S_A-J_, red coils represent helical structures labeled H_1-2_ and gaps indicate coil regions. Black boxes indicate the amino acids that were ≥80% conserved between FABP sequences included in the MSA (figure 6).

The configuration of secondary structural elements in the EBP(Ec) amino acid sequence was investigated using *ab intio* prediction methods. Five freely available, web server algorithms: YASPIN [57], SPINEX [58], NetSurfP [59], PSIPRED [60] and PHD [61] were used for structure prediction, from which a consensus was obtained. The consensus predicted 10 β-strands linked by short loop regions, with two N-terminal proximal α-helices situated between strands A and B (Figure 7). This positioning of the predicted secondary structural elements in the EBP(Ec) sequence is consistent with the conserved structure of FABPs; a 10 stranded antiparallel β-barrel with two α-helices which cap one end of the barrel to enclose the ligand-binding site [87], [88].

The positions of the highly conserved residues (≥80%) identified by MSA were mapped onto the secondary structure prediction (Figure 7 black boxes), to determine whether they corresponded to particular features. Three of the conserved amino acids are glycine residues that are predicted to be situated within turns, between S_B_–S_C_, S_D_–S_E_ and S_F_–S_G_. Glycine is common in protein loops and turns as the absence of a side chain allows for flexibility [91], [92]. The turns in EBP(Ec) are predicted to be tight, 2–3 amino acids in length, therefore small flexible amino acids are likely to be conserved. The majority of the other highly conserved residues are predicted to lie within the β-strands and previous analysis of FABP crystal structures suggests that they are likely to contribute to stability of the tertiary structure and formation of the hydrophobic pocket [87].

**Figure 8:**
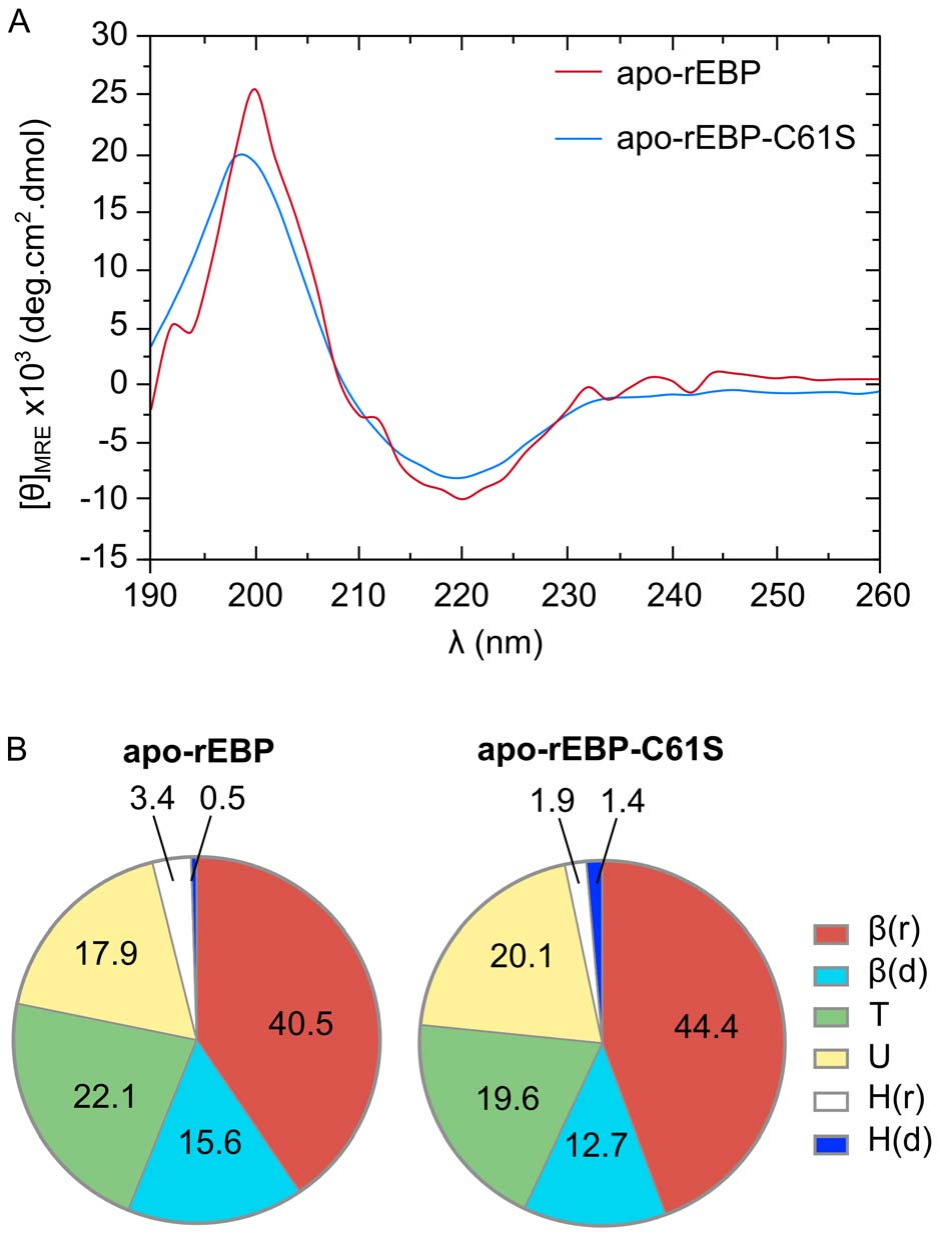
CD spectra of recombinant apo-rEBP and apo-rEBP-C61S. Spectra were collected in the presence of 5 mM phosphate, pH 7.5 at 18°C. **A**. CD spectra of apo-rEBP is shown in red and apo-rEBP-C61S is shown in blue. Predictions of secondary structural elements in apo-rEBP and apo-rEBP-C61S. **B**. The Δε data derived from CD spectra were analyzed using CDPro and percentage averages of each element, predicted by CDSSTR and CONTINLL, are shown for the SMP53 protein reference set. Regular helix - H(r), distorted helix - H(d), regular sheet - β(r), distorted sheet – β(d), turns - T and unordered – U.

In order to attempt to experimentally validate the *ab initio* secondary structure predictions, circular dichroism spectroscopy (CD) measurements were made on both recombinant apo-EBP and apo-EBP-C61S. No significant differences were observed between the mutant and wt CD spectra (Figure 8.A and Figure S5), suggesting that the mutation causes little or no perturbation to secondary structure. Both spectra exhibited characteristics of proteins dominated by β-sheet structures (Figure 8.A), with local maxima and minima centered at 195 nm and 220 nm respectively [62]. The CD data were then input into a suite of algorithms provided by CDPro [64], [65], [66], [67], [68], for secondary structure prediction for comparison to the *ab initio* methods.

CDPro predicts the proportions of secondary structural elements based on the comparison of spectral features to a reference protein set. There are six secondary elements recognized that consist of α-helix and β-strand, which are both subdivided into regular and distorted structures, where <4 residues and <2 residues considered distorted helix or strand respectively, and coil which encompasses both turns and disordered structure [64], [65], [66], [67], [68]. The predicted proportions of each secondary structural element were consistent between apo-rEBP and apo-rEBP-C61S (Figure 8.B). The total β-strand content was predicted to be 56–57%, which was equivalent to the 55.1% from the *ab initio* consensus prediction (Table 3). However, the CD data resulted in a lower predicted proportion of helix 3.3–3.7%, compared to an average of 10.8% from the *ab initio* methods, which was offset by the prediction of a higher percentage of coil (Table 3). The discrepancy in the prediction of helical content between the two methods may reflect structural flexibility in this region of the protein, resulting in an increased prediction of disordered residues during dynamic solution based CD measurements. This is consistent with the observed lack of definition in the helix motifs of previous apo-FABP solution-state structures [89], [93]. Therefore, the CD data are considered to be consistent with the *ab initio* secondary structure prediction and amino acid sequence homology analyses, suggesting that EBP(Ec) is a member of the FABP family.

**Table 3:** Proportions of secondary structural elements predicted by *ab initio* methods and CD.

Secondary structure prediction method	% Helix	% Strand	% Coil
*Ab initio* consensus: EBP	10.8	55.1	29.0
CD: EBP	3.7	56.0	40.5
CD: EBPC61S	3.3	57.0	39.5

## Conclusion

This report provides evidence for the existence of a novel carotenoid-binding protein (CBP) present in the gonad of the New Zealand sea urchin *E. chloroticus*. The CBP was found to bind mainly 9′-*cis*-echinenone and has therefore been called an echinenone-binding protein (EBP) and appears to be involved in selective accumulation of echinenone in the gonad. The existence of the EBP was supported by analysis of a recently reported *E. chloroticus* transcriptome [53] and by identification of an EBP homologue in the *S. purpuratus* genome [46] that has not previously been identified, and thus provides annotation of these genes.

The EBP and C61S mutant, which exhibited enhanced solubility, were produced recombinantly. Analysis of the mutant and wild type recombinant proteins by CD, together with *ab initio* secondary structure prediction, suggested that EBP adopts a 10 β-stranded structure with a low helical content. The predicted arrangement of the secondary structural elements is consistent with the antiparallel β-barrel topology that is characteristic of the FABP family.

Although it has been previously hypothesized that FABP may bind carotenoids, aiding digestion and absorption [94], to our knowledge none have been characterized. Therefore the EBP may represent the first report of a FABP in complex with a carotenoid.

The highly conserved tertiary fold of the FABP family provides some tantalizing clues to the structure of EBP and ligand binding. However, these results now prompt the determination of the structure of this novel CBP. A holo-EBP structure elucidating the interactions of the carotenoid in the FABP pocket may suggest a mechanism by which the protein achieves the selective accumulation of echinenone in the sea urchin gonad.

## Supporting Information

Figure S1 **Mass spectrometry sequence coverage of EBP variants.** EBP, apo-rEBp and apo-rEBP-C61S were subjected to in-gel digestion with trypsin (T). In addition apo-rEBP-C61S was also subjected to in-gel digestion with Glu-C endoproteinase (GC). The peptides were analyzed by MALDI-TOF/TOF mass spectrometry and identified by a Mascot search against a user database containing the predicted EBP(Ec) amino acid sequence. The peptides identified for each protein were mapped onto the EBP(Ec) sequence and are indicated by the colored lines beneath the sequence. The position of the mutation, C61S, is indicated in large bold type and the identity of the amino acid at position 61 is indicated for each peptide.
(TIFF)

Figure S2 **EBP(Ec) cDNA sequence.** The cDNA sequence of the EBP was obtained by searching the *S. purpuratus* sequence against the *E. chloroticus* transcriptome *de novo* assembly. The cDNA sequence is shown above the amino acid translation. Initiator and terminator codons are shown in red text and the ORF in black text. Parts of the 5′ and 3′ UTRs, flanking the ORF are shown in blue text and the mass spectrometry matched peptide is shown in green text.
(TIFF)

Figure S3 **DNA sequencing confirmation of C61S site directed mutagenesis.** DNA sequencing of plasmid isolates containing either the EBP (A) or the EBP-C61S (B) sequence was performed by Genetic Analysis Services, University of Otago, Dunedin, New Zealand. Sequencing results were viewed in 4Peaks; red circles indicate the location of the codon change. Only nucleotides 156–201 are shown.
(TIFF)

Figure S4 **Apo-rEBP-C61S associates with 9′-** ***cis*** **-echinenone** ***in vitro*** **.** A 500 µL aliquot of each of apo-rEBP, apo-rEBP-C61S and BSA at a concentration of 2 mg.mL^−1^ were incubated for 1 h at 37°C with 10 and 100 nmol of HPLC-purified 9′-*cis*-echinenone in 10% v/v acetone. Unbound carotenoid was removed by dialysis in 10 kDa MWCO tubing overnight against 50 mM dibasic sodium phosphate pH 8.0, followed by centrifuging at 13,000 *g* for 10 min. The supernatant was removed and photographed against a white background, under indoor light conditions, using a Cannon PowerShot A2500 digital camera with flash. Scale bar represents 10 mm.
(TIFF)

Figure S5 **CD spectra of recombinant apo-EBP and apo-EBP-C61S.** Spectra were collected in the presence of 5 mM phosphate, pH 7.5 at 18°C. Each spectrum is the average of 5 datasets collected with an average baseline subtracted. Error bars are ± standard deviation (SD) calculated by √(SD_sample_^2^ + SD_baseline_^2^}. Apo-EBP is shown in black and apo-EBP-C61S mutant shown in red.
(TIFF)

Table S1 **MALDI TOF/TOF mass spectrometry peptides sequence coverage of EBP, apo-rEBP and apo-rEBP-C61S.**
(DOCX)

Table S2 **Water local pairwise sequence alignment summaries of FABP sequences against** ***E. chloroticus*** **EBP.**
(DOCX)
