# Supplementary figures and images for: A Novel Fatty Acid-Binding Protein-Like Carotenoid-Binding Protein from the Gonad of the New Zealand Sea Urchin Evechinus chloroticus
Source: PLoS One. 2014 Sep 5;9(9):e106465. doi: 10.1371/journal.pone.0106465 (PMC4156332; doi:10.1371/journal.pone.0106465)

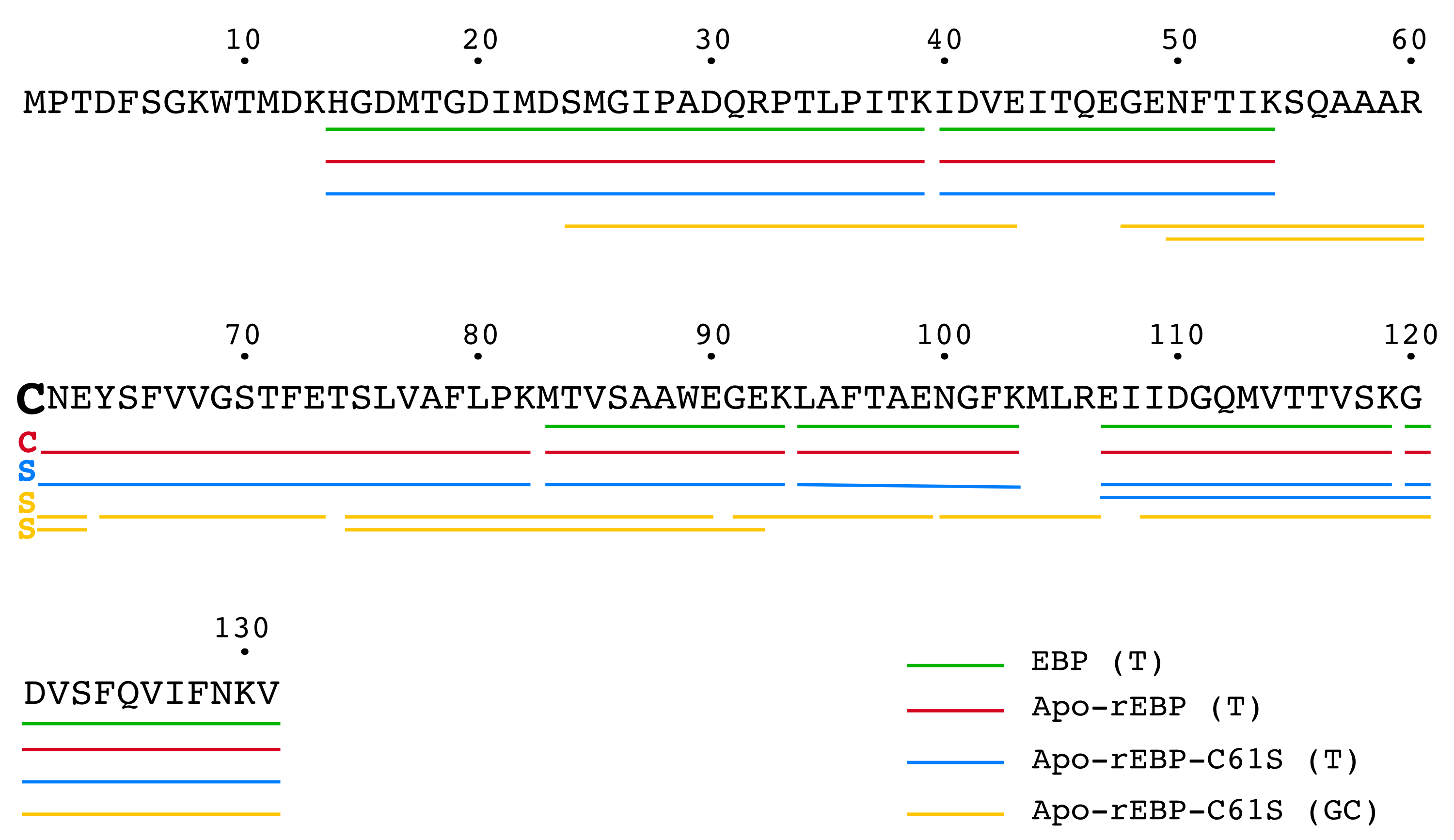

Supplement: Figure S1 — Mass spectrometry sequence coverage of EBP variants. EBP, apo-rEBp and apo-rEBP-C61S were subjected to in-gel digestion with trypsin (T). In addition apo-rEBP-C61S was also subjected to in-gel digestion with Glu-C endoproteinase (GC). The peptides were analyzed by MALDI-TOF/TOF mass spectrometry and identified by a Mascot search against a user database containing the predicted EBP(Ec) amino acid sequence. The peptides identified for each protein were mapped onto the EBP(Ec) sequence and are indicated by the colored lines beneath the sequence. The position of the mutation, C61S, is indicated in large bold type and the identity of the amino acid at position 61 is indicated for each peptide. (TIFF) [file pone.0106465.s001.tiff]

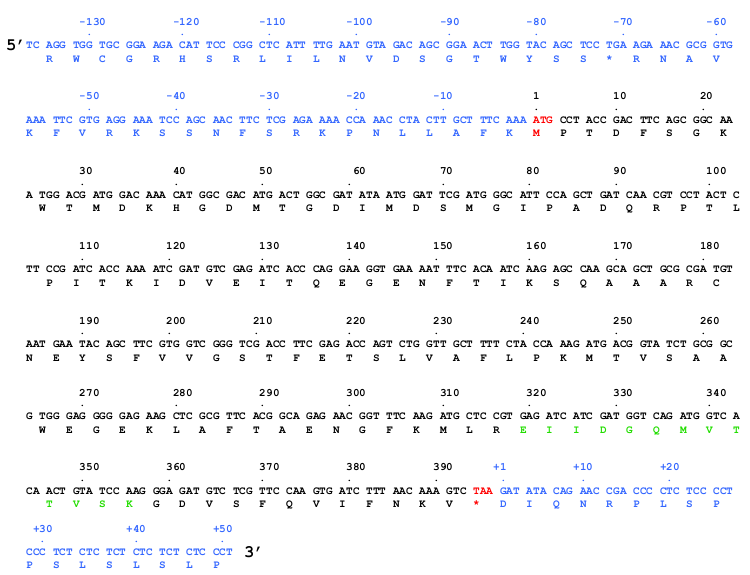

Supplement: Figure S2 — EBP(Ec) cDNA sequence. The cDNA sequence of the EBP was obtained by searching the S. purpuratus sequence against the E. chloroticus transcriptome de novo assembly. The cDNA sequence is shown above the amino acid translation. Initiator and terminator codons are shown in red text and the ORF in black text. Parts of the 5′ and 3′ UTRs, flanking the ORF are shown in blue text and the mass spectrometry matched peptide is shown in green text. (TIFF) [file pone.0106465.s002.tiff]

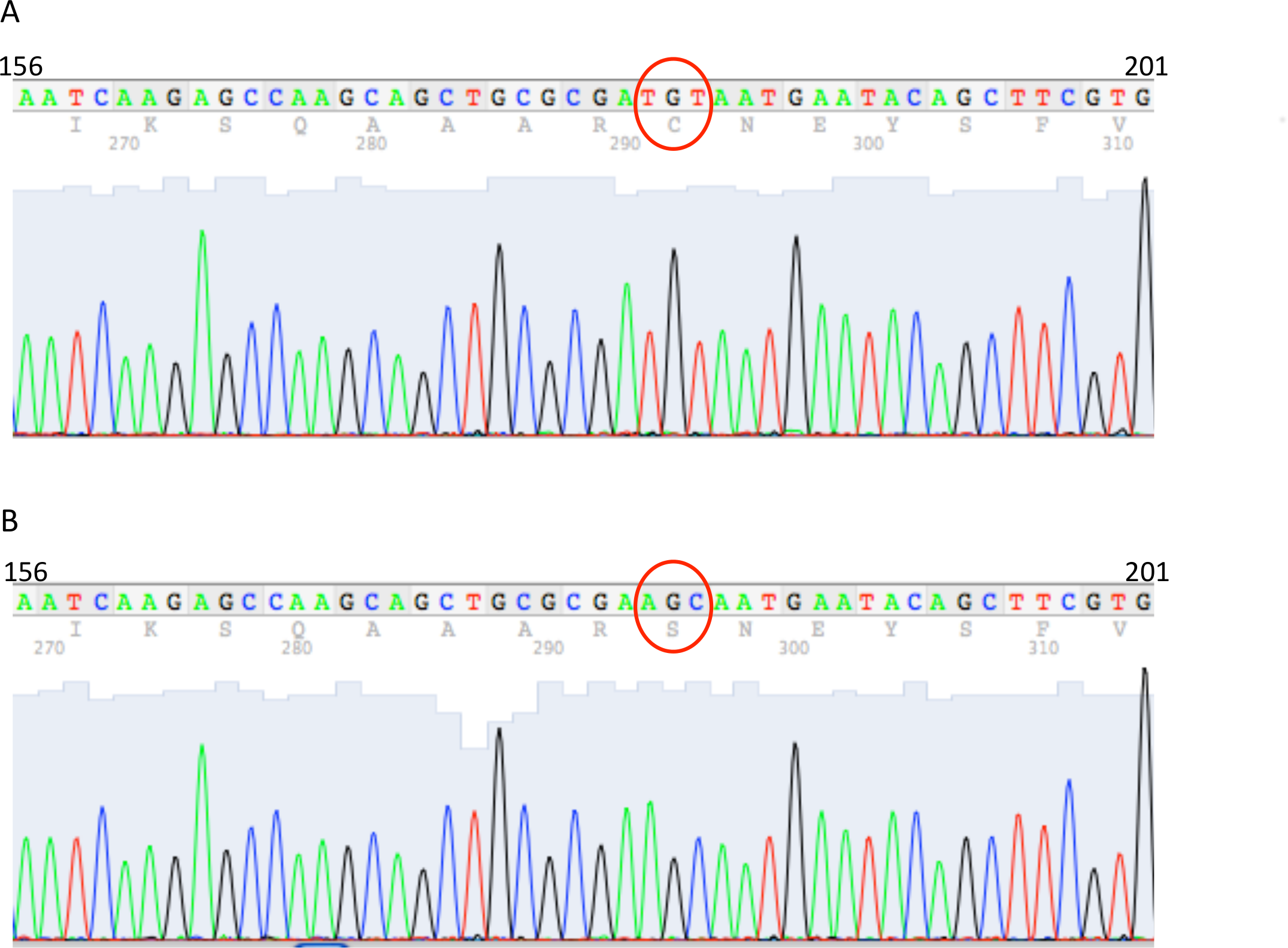

Supplement: Figure S3 — DNA sequencing confirmation of C61S site directed mutagenesis. DNA sequencing of plasmid isolates containing either the EBP (A) or the EBP-C61S (B) sequence was performed by Genetic Analysis Services, University of Otago, Dunedin, New Zealand. Sequencing results were viewed in 4Peaks; red circles indicate the location of the codon change. Only nucleotides 156–201 are shown. (TIFF) [file pone.0106465.s003.tiff]

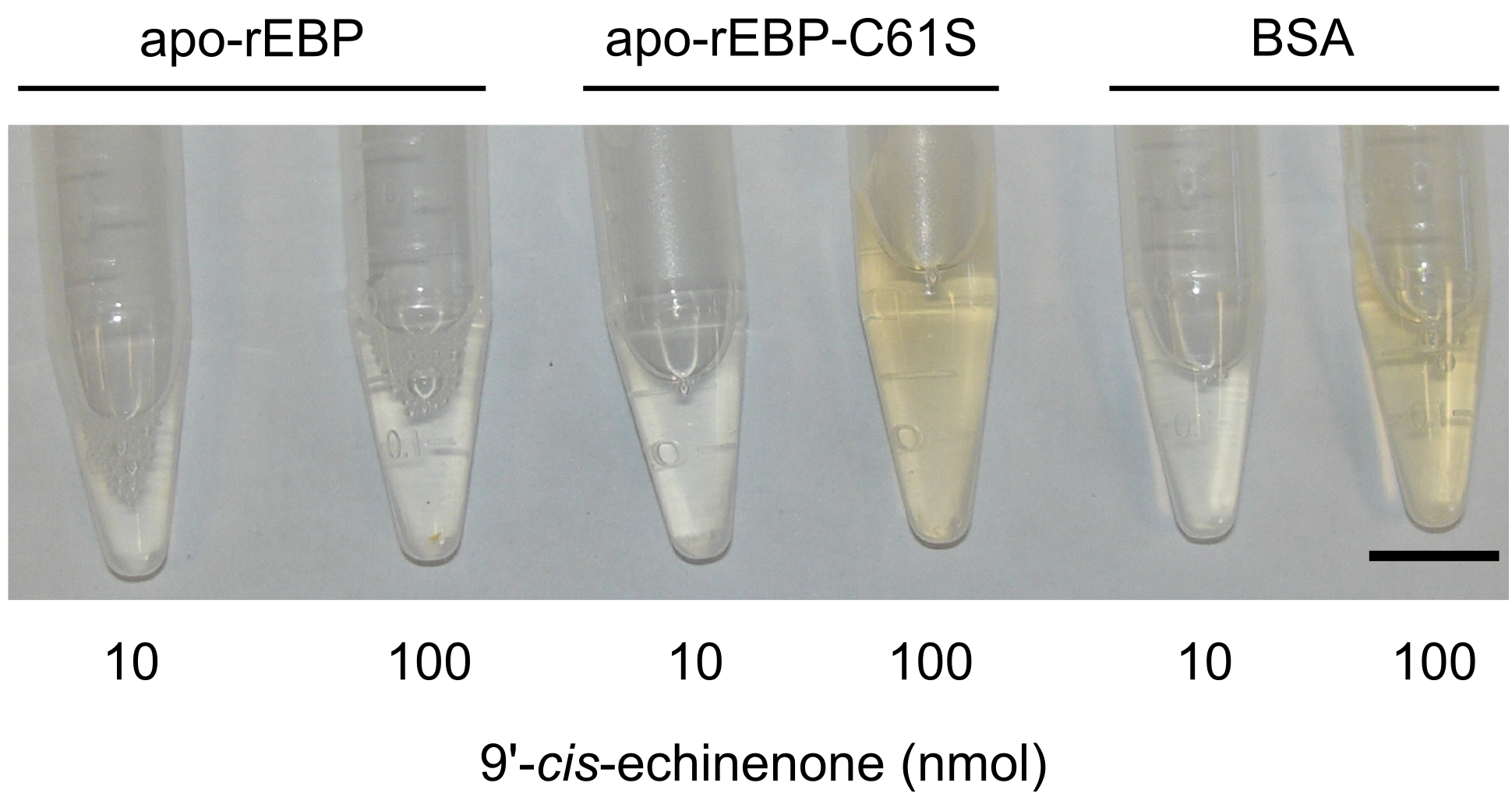

Supplement: Figure S4 — Apo-rEBP-C61S associates with 9′- cis -echinenone in vitro . A 500 µL aliquot of each of apo-rEBP, apo-rEBP-C61S and BSA at a concentration of 2 mg.mL−1 were incubated for 1 h at 37°C with 10 and 100 nmol of HPLC-purified 9′-cis-echinenone in 10% v/v acetone. Unbound carotenoid was removed by dialysis in 10 kDa MWCO tubing overnight against 50 mM dibasic sodium phosphate pH 8.0, followed by centrifuging at 13,000 g for 10 min. The supernatant was removed and photographed against a white background, under indoor light conditions, using a Cannon PowerShot A2500 digital camera with flash. Scale bar represents 10 mm. (TIFF) [file pone.0106465.s004.tiff]

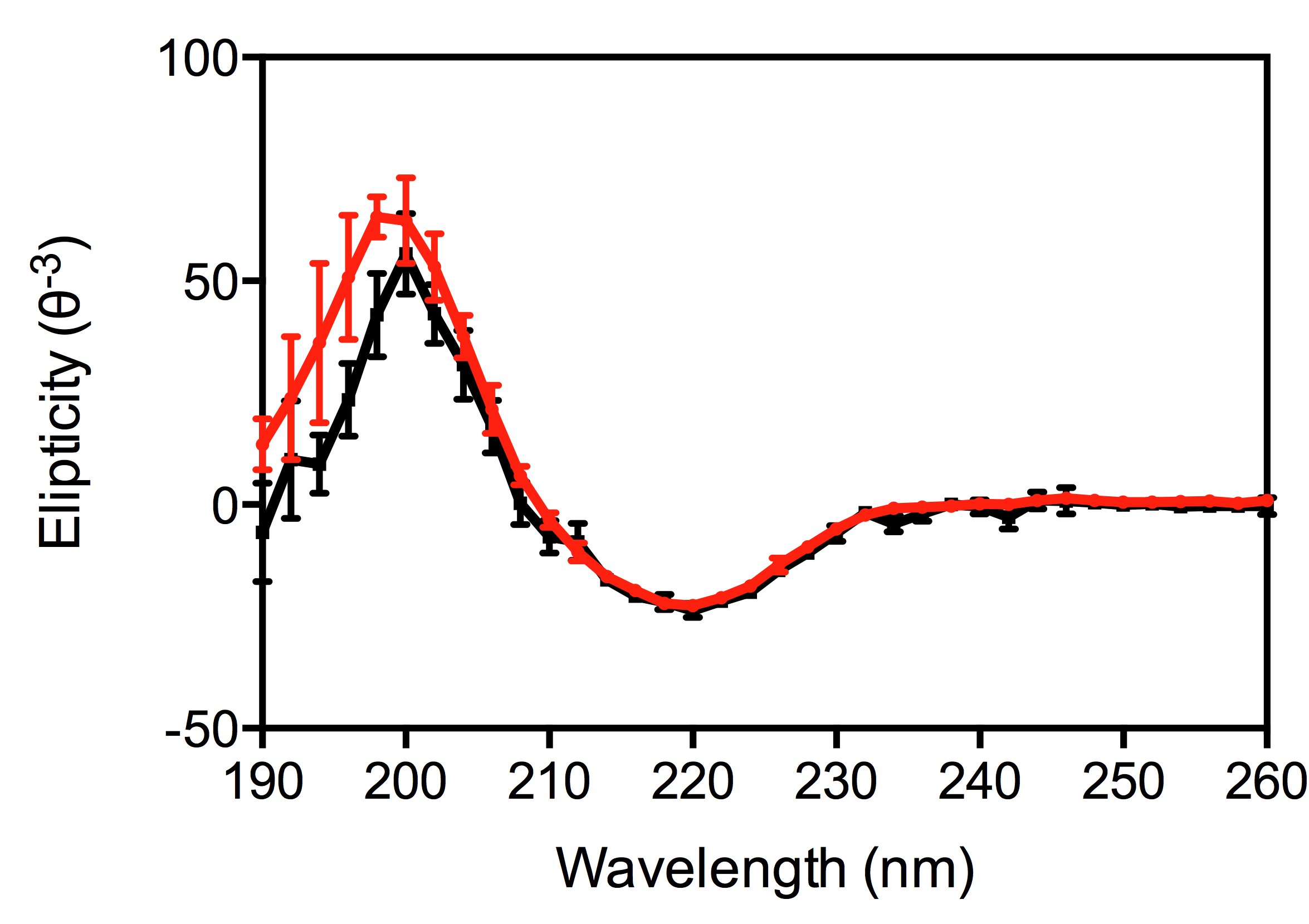

Supplement: Figure S5 — CD spectra of recombinant apo-EBP and apo-EBP-C61S. Spectra were collected in the presence of 5 mM phosphate, pH 7.5 at 18°C. Each spectrum is the average of 5 datasets collected with an average baseline subtracted. Error bars are ± standard deviation (SD) calculated by √(SDsample 2 + SDbaseline 2}. Apo-EBP is shown in black and apo-EBP-C61S mutant shown in red. (TIFF) [file pone.0106465.s005.tiff]
