# Supplementary material for: A Novel Fatty Acid-Binding Protein-Like Carotenoid-Binding Protein from the Gonad of the New Zealand Sea Urchin Evechinus chloroticus
Source: PLoS One. 2014 Sep 5;9(9):e106465. doi: 10.1371/journal.pone.0106465 (PMC4156332; doi:10.1371/journal.pone.0106465)
Supplement: Table S1 — MALDI TOF/TOF mass spectrometry peptides sequence coverage of EBP, apo-rEBP and apo-rEBP-C61S. (DOCX) [file pone.0106465.s006.docx]

**Supplementary tables**

Table S1. MALDI TOF/TOF mass spectrometry peptides sequence coverage of EBP, apo-rEBP and apo-rEBP-C61S.

| Peptide | Modifications^a^ | Enzyme (T/GC)^b^ | Sequence Position (1-131) | EBP Score^c^ | Apo-rEBP Score | Apo-rEBP-C61S Score |
| --- | --- | --- | --- | --- | --- | --- |
| HGDMTGDIMDSMGIPADQRPTLPITK |  | T | 14-39 | 120 | 76 | 131 |
| HGDMTGDIMDSMGIPADQRPTLPITK | +Oxidation(M) | T | 14-39 | 84 | 80 | 92 |
| HGDMTGDIMDSMGIPADQRPTLPITK | +2Oxidation(M) | T | 14-39 | 61 | 56 | 38 |
| HGDMTGDIMDSMGIPADQRPTLPITK | +3Oxidation(M) | T | 14-39 | 48 | 60 | x^d^ |
| SMGIPADQRPTLPITKIDVE |  | GC | 24-43 | -^d^ | - | 104 |
| IDVEITQEGENFTIK |  | T | 40-54 | 136 | 107 | 117 |
| GENFTIKSQAAARSNE |  | GC | 48-63 | - | - | 67 |
| NFTIKSQAAARSNE |  | GC | 50-63 | - | - | 99 |
| SNEYSFVVGSTFETSLVAFLPK |  | T | 61-83 | - | - | 148 |
| CNEYSFVVGSTFETSLVAFLPK | +Carbamidomethyl(C) | T | 61-83 | x | 98 | - |
| YSFVVGSTFE |  | GC | 64-73 | - | - | 53 |
| TSLVAFLPKMTVSAAWE |  | GC | 74-90 | - | - | 90 |
| TSLVAFLPKMTVSAAWE | +Oxidation (M) | GC | 74-90 | - | - | 50 |
| TSLVAFLPKMTVSAAWEGE |  | GC | 74-92 | - | - | 79 |
| MTVSAAWEGEK |  | T | 83-93 | 80 | 78 | 90 |
| MTVSAAWEGEK | +Oxidation(M) | T | 83-93 | 64 | 45 | x |
| GEKLAFTAE |  | GC | 91-99 | - | - | 26 |
| LAFTAENGFK |  | T | 94-103 | 91 | 84 | 87 |
| NGFKMLRE |  | GC | 100-107 | - | - | 23 |
| EIIDGQMVTTVSK |  | T | 107-119 | 81 | 94 | 74 |
| EIIDGQMVTTVSKGDVSFQVIFNKV |  | T | 107-131 | x | x | 51 |
| IIDGQMVTTVSKGDVSFQVIFNKV |  | GC | 108-131 | - | - | 112 |
| IIDGQMVTTVSKGDVSFQVIFNKV | +Oxidation (M) | GC | 108-131 | - | - | 75 |
| GDVSFQVIFNK |  | T | 120-130 | 67 | 68 | x |
| GDVSFQVIFNKV |  | T | 120-131 | 74 | 70 | 84 |

^a^ Modified amino acid(s) are indicated in parentheses and are underlined in the peptide sequence.

^b^ Trypsin (T) used for digestion of all three protein variants and Glu-C-endoproteinase (GC) for the digestion of rEBP-C61S only to gain sufficient coverage of mutation site.

^c^ Individual ion scores >14 indicate identity, or extensive homology (p<0.05).

^d^ x indicates peptide was not found and horizontal dash indicates not applicable.
