# Supplementary material for: A Novel Fatty Acid-Binding Protein-Like Carotenoid-Binding Protein from the Gonad of the New Zealand Sea Urchin Evechinus chloroticus
Source: PLoS One. 2014 Sep 5;9(9):e106465. doi: 10.1371/journal.pone.0106465 (PMC4156332; doi:10.1371/journal.pone.0106465)
Supplement: Table S2 — Water local pairwise sequence alignment summaries of FABP sequences against E. chloroticus EBP. (DOCX) [file pone.0106465.s007.docx]

Table S2. Water local pairwise sequence alignment summaries of

FABP sequences against *E. chloroticus* EBP.

| Query sequence | % Identity | % Similarity | % Gaps | Alignment length (amino acids) | Alignment score |
| --- | --- | --- | --- | --- | --- |
| EBP *S. purpuratus* | 65.3 | 84.7 | 0.0 | 118 | 418.0 |
| FABP2 *Rattus norvegicus* | 29.8 | 48.1 | 5.3 | 131 | 118.5 |
| cRABP *Homo sapiens* | 28.8 | 45.5 | 9.8 | 132 | 117.5 |
| RBP1 *Homo sapiens* | 26.1 | 43.7 | 13.4 | 142 | 108.0 |
| FABP1 *Rattus norvegicus* | 21.1 | 38.8 | 1.6 | 128 | 80 |
| ALBP *Rattus norvegicus* | 22.4 | 40.3 | 16.4 | 134 | 71.5 |
